# Investigating themes in hearing quality of life with user-nominated goals on the Glasgow Hearing Aid Benefit Profile (GHABP)

**DOI:** 10.1186/s41687-025-00886-1

**Published:** 2025-07-01

**Authors:** Avivah J. Wang, Grace Strong, Kayla W. Kilpatrick, Sherri L. Smith, Theresa Coles

**Affiliations:** 1 Duke University School of Medicine, NC Durham, USA; 2 Center for Health Measurement, Department of Population Health Sciences, Duke University, Durham, NC USA; 3 Department of Biostatistics and Bioinformatics, Duke University School of Medicine, Durham, NC USA; 4 Department of Head & Neck Surgery and Communication Sciences, Duke University School of Medicine, Durham, NC USA; 5 Department of Population Health Sciences, Duke University School of Medicine, Durham, NC USA; 6 Center for Aging, Duke University School of Medicine, DUMC 3887, Durham, NC 27710 USA

**Keywords:** Patient-reported outcome measures, Questionnaire, Age-related hearing loss, Hearing aids, Audiology, Patient goals

## Abstract

**Background:**

The Glasgow Hearing Aid Benefit Profile (GHABP) is a patient-reported outcome measure (PROM) that was developed for the assessment of hearing aid efficacy using standard goals and user-nominated goals. The objective of this study was to describe user-nominated hearing goals to determine themes that are not currently being captured by the standard goals and that could improve comprehensive assessment of hearing quality of life with the GHABP for use in clinical trials.

**Methodology:**

We conducted a secondary analysis of a clinical trial at two tertiary care institutions. Adults ≥ 50 years of age with hearing loss completed the GHABP before treatment, including the portion where they provided up to two user-nominated goals for situations where hearing was personally challenging to them. We then categorized these goals into themes.

**Results:**

A total of 262 participants completed the standard GHABP and provided a total of 501 user-nominated goals. Common themes were having a conversation with several people in a group (80/501, 16.0%), hearing in background noise (73/501, 14.6%), and listening when unable to see the speaker’s mouth (57/501, 11.4%).

**Conclusions:**

Themes of listening in background noise and listening when unable to see the speaker’s mouth are very important to many individuals with hearing loss. Expanding PROMs to include these goals may improve patient-centeredness of clinical trial and clinical care outcomes tracking.

## Background

Hearing loss is prevalent in older adults, affecting approximately 28.5% of adults in the fifth decade of life and increasing to more than two-thirds of adults 70 years of age and older [1]. Hearing loss can be associated with significant morbidity, including depression [2], social isolation [3], and poorer cognitive function [4]. A study analyzing the impact of hearing aids and cochlear implants found that treatment for hearing loss led to significantly increased mental health quality of life, as measured by the Mental Component Summary score of the Short-Form Health Survey (SF-36) [5].

Hearing aids are the primary intervention for age-related hearing loss, and treatment of hearing loss can slow cognitive decline [6, 7]. Hearing aids are programmed by audiologists to maximize patient outcomes and quality of life. It is therefore important to understand the specific hearing challenges to measure the efficacy of treatment with hearing aids and better mitigate the negative effects of hearing loss and support patient-centered care.

The Glasgow Hearing Aid Benefit Profile (GHABP), published in 1999, is a questionnaire used for assessment of efficacy of hearing aid use [8]. The GHABP asks patients to record their level of difficulty with hearing and impact on quality of life in four pre-determined scenarios, followed by the opportunity to nominate unique situations/goals. It has been shown that patient-centered care, which emphasizes considering patients’ perspectives in their treatment, improves overall well-being and outcomes [9, 10]. Collecting and understanding patient goals for hearing, and clinical care in general, is therefore vitally important. Because the GHABP was developed more than two decades ago for use in clinical care, it is possible that the predetermined scenarios assessed may be less applicable in a clinical trial setting, or outdated due to changes in technology, communication practices, and the COVID-19 pandemic.

Qualitative studies in patients with hearing loss have identified several domains that are important to patients, including communication in-person and with technologies such as telephones, recognizing environmental sounds, increased cognitive effort spent on listening, and difficulties in new and existing social relationships, among others [11]. These may be important aspects of patients’ experiences with hearing loss when evaluating the impact of hearing aids on quality of life that are not standard in the current form of the GHABP.

Our study aims to assess user-nominated scenarios to determine themes among users of one or two hearing aids in a randomized clinical trial (NCT04739436). Using GHABP in a clinical trial may raise different goals for patients than what is considered in clinical care. The rationale for this study is to describe hearing goals specifically in the clinical trial context and to address potential changes in patients’ hearing goals since the GHABP was originally developed.

## Methods

This study is a secondary data analysis from a randomized clinical trial. The primary study was a randomized, parallel-group, two-phase clinical trial comparing the hearing-aid benefit of unilateral and bilateral fittings of hearing aids [12]. Participants provided written informed consent. Participants were provided $50 remuneration and a parking voucher for completing the baseline visit. This study was approved by Duke Institutional Review Board (Pro00106077).

### Participants

Adults ≥ 50 years of age with age-related, mild-to-moderate hearing loss with no prior history of hearing aid use who were pursuing amplification and provided goals on the GHABP were eligible for this secondary data analysis. Inclusion criteria details for the parent study are presented in the Appendix.

### Recruitment

Potentially eligible participants were identified from audiology clinics at 2 sites. Those who met inclusion and exclusion criteria for the parent study were approached by a member of the clinical research team to share details about the study and assess their interest in participating. If interested, then participants completed written informed consent.

### Procedures

Questionnaires were completed electronically through REDCap at baseline [13, 14]. The principal investigators and non-statistical co-investigators were blinded to treatment assignment, while clinical research coordinators and study audiologists were not blinded. Participants were randomly assigned to treatment via a random-number generator at a 1:1 ratio, stratified by clinical site. Data for this secondary analysis study are from baseline assessments only.

### Sample size

Sample size was determined based on the primary study [12, 15] to detect differences on the Abbreviated Profile of Hearing Aid Benefit (APHAB) scores by study arm [16].

### Data collection

Demographic and background characteristics of participants, as well as unaided auditory-based performance metrics, and patient-reported outcome measure scores were collected at the baseline visit.

### Measures

The GHABP is a patient-reported questionnaire developed to evaluate individual hearing difficulties. There are four pre-specified goals of the GHABP: (1) listening to the television when the volume is adjusted for others, (2) having a conversation with one person in quiet, (3) having a conversation on a busy street or in a shop, and (4) having a conversation with several people in a group. In the original GHABP, participants are invited to nominate 4 additional goals of their choosing. In contrast, in this study, participants were invited to nominate only two additional goals, with the original GHABP language otherwise retained: “We have dealt with some of the situations which in our experience can lead to difficulty with hearing. What we would like you to do is to nominate up to two new situations in which it is important for you as an individual to be able to hear as well as possible.”

For each standard goal, participants are asked 3 follow-up questions:

1. Does this situation happen in your life? [yes, no]
2. How much difficulty do you have in this situation? [5-point scale: 1- ‘no difficulty’ to 5- ‘cannot manage at all’]
3. How much does any difficulty in this situation worry, annoy or upset you? [5-point scale: 1- ‘not at all’ to 5- ’very much indeed’]

**Fig. 1:**
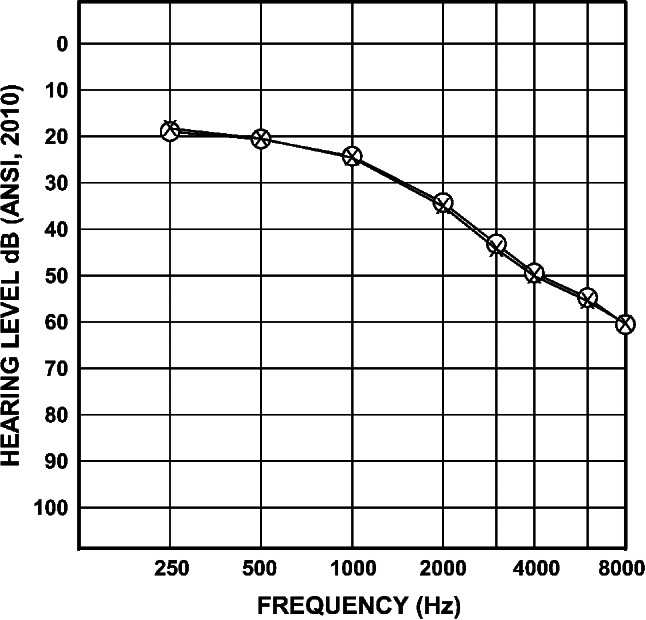
Mean audiogram of the participants; circles = right ear thresholds, x = left ear thresholds

Questions 2 and 3 are only asked for the standard goals if the individual responds “yes” to question 1. Participants are then asked questions 2 and 3 for each of their user-nominated goals. Higher total scores on the GHABP indicate greater difficulty with hearing. Audiograms were obtained at pre-study and initial baseline study visits (Fig. 1). Baseline study visit audiogram data were used if available for any given frequency; otherwise, data from the screening clinical audiology visit prior to study enrollment were used.

### Analysis

Descriptive statistics were tabulated for participant characteristics, and audiograms from their standard clinic visit were captured. Coding was conducted in two stages. In stage 1, deidentified user-nominated GHABP goals were output and reviewed (AJW, TC). Draft codes were deductively and inductively developed collaboratively (AJW, TC) to thematically classify user-nominated goals (Table 1). Inductive codes were developed after reading through all open-ended goals while minimizing assumptions about the goal and taking what was written by the patient without additional interpretation or assumption. After coding began, some codes were combined or separated for clarity. Then, one coder (AJW) coded all user-nominated goals using the draft codebook. In stage 2, a third research team member (GS) conducted blind double-coding of 15% of the codes. Using a randomization website, https://www.random.org/, 15% of the participants’ goals were randomly selecting for second reviewer coding. An a-priori threshold of 90% matching codes between reviewers was set to ensure consistency of code application. The threshold was not met; the team (AJW, GS, TC) met to discuss code definitions and application of codes. The codebook was updated, and a research team member (GS) reviewed and updated all user-nominated codes.

**Table 1:** Codebook structure

Code	Description	Deductive	Inductive
Falls into standard GHABP 1	Per GHABP: “Listening to the television with other family or friends when the volume is adjusted to suit other people.”	x	
Falls into standard GHABP 2	Per GHABP: “Having a conversation with one other person when there is no background noise.”	x	
Falls into standard GHABP 3	Per GHABP: “Carrying on a conversation in a busy street or shop.” ***Assumptions***: Includes situations in restaurants and other busy locations. Private events in a home (i.e. family gatherings, etc. would not be included in this code.	x	
Falls into standard GHABP 4	Per GHABP: “Having a conversation with several people in a group.” ***Assumptions***: Include any busy, public situation that cannot be readily controlled by the participant. Private events in a home (i.e. family gatherings, etc. would be included in this code.	x	
Listening when participant cannot see the speakers’ mouth	The act of comprehending spoken language without visual cues from the speaker’s lip movements or facial expressions. For this code, the participant and speaker are in the general vicinity of each other. For example, a participant talking to someone who is in another room of their home, or the participant is driving in their car and talking to a passenger in the backseat. Not included in this definition: talking to someone on the phone, listening to a podcast, etc.		x
Using technology to listen to other people or content (Zoom, speaker phone, telephone, headphones)	The use of electronic devices and platforms to receive auditory information from others or from media content. For example, participating in a Zoom meeting, listening to a lecture (live or recorded), or having a phone conversation. Also included in this category is listening to a radio. Listening to music using technology may also be included under this code.		x
Background noise causes challenges hearing a subject	Difficulty in understanding spoken words due to interference from surrounding environmental sounds. For example, having a conversation with someone at a party, or during a noisy family gathering. Struggling to hear a conversation in a busy café/restaurant may be included here, but it would also be included in GHABP 3.		x
Listening to performances/lectures	The act of attending a live presentation, including musical performances, theatrical productions, or educational lectures. Church services or sermons are included in this definition.		x
Pitch	The perception of the frequency of a sound, which determines how high or low it sounds. For example, the participant having a conversation with their spouse who has a high-pitched voice.		x
Music	Listening to organized sound patterns that are typically melodic, harmonic, and rhythmic. For example, listening to the participant’s favorite song. Music may be live or recorded. This can include listening to music using a radio or another technology platform.		x
Quiet/soft speech	Speech that is delivered at a low volume, making it more challenging to hear and understand. For example, a person whispering or speaking softly in a quiet room.		x
Not specific enough to be actionable	The goal provided lacks sufficient detail therefore a code was not assigned.	x	
Other: Unable to be categorized	The goal provided is specific enough to be actionable but unable to be categorized because it does not fit into any of the codes listed.	x	
More than one goal listed	The goal provided could be categorized into multiple codes.	x	

User-nominated goals could be coded with more than one code. For example, a complex goal such as “listening to family members in another room while cooking” would be classified under “listening when participant cannot see the speaker’s mouth” and “background noise causes challenges hearing a subject.” Because this goal would fall under multiple codes, it would additionally be coded as “more than one goal listed.”

After coding was complete, the number of user-nominated goals categorized by each code was tabulated.

## Results

Of the 275 participants in the primary study, 262 participants—with a total of 501 user-nominated goals—were included in this secondary analysis. Thirteen participants from the primary study were excluded for this analysis because they did not complete the user-nominated goal portion of the GHABP at their initial visit or provided answers such as “none” or “not applicable” for both user-nominated questions. The mean age of included participants was 70.8 years (SD: 7.9 years). The majority of participants were female (143/262, 54.6%), Caucasian or White (248/262, 94.7%), and not Hispanic or Latino (249/262, 95.0%). Most participants were highly educated, with 137/262 (52.3%) holding a graduate degree, and most participants lived with a spouse (173/262, 66.0%). A more detailed description of the study cohort is in Table 2.

**Table 2:** Description of participants in this secondary data analysis

Description	*N* (%)
Total participants	262 (100%)
Age	
Mean (SD)	70.8 (7.9)
Median (Range)	71.0 (50.0–94.0)
Sex	
Female	143 (54.6%)
Male	119 (45.4%)
Race	
American Indian or Alaska Native	*
Black/African American	*
Caucasian/White	248 (94.7%)
More than one race	*
Declined	*
Ethnicity	
Hispanic or Latino	*
Not Hispanic or Latino	249 (95.0%)
Declined	*
Education	
Less than high school	2 (0.8%)
High School	7 (2.7%)
Some College	29 (11.1%)
4-year Degree	85 (32.4%)
Graduate Degree	137 (52.3%)
Other	2 (0.8%)
Living Arrangements	
Alone	50 (19.1%)
With Spouse	173 (66.0%)
With Spouse and Others	25 (9.5%)
Other	14 (5.3%)
Site	
Duke	229 (87.4%)
Vanderbilt	33 (12.6%)

* Counts < 10; exact numbers are not reported to prevent patient identifiability

Participants’ aggregated responses to the four standard GHABP goal questions are presented in Table 3. Each of the standard goal situations was encountered in at least 80% of participants’ lives. Participants indicated the most difficulty in standard GHABP goal 1 (listening to the TV when the volume is adjusted for others) and goal 3 (having a conversation on a busy street of in a shop), with a mean response of 3.0 on a 1 to 5 scale for both, with higher scores indicating greater difficulty. Participants indicated they were most worried, annoyed, or upset by their difficulty in standard goal 4 (having a conversation with several people in a group), with a mean response of 2.8 on a 1 to 5 scale, with higher scores indicating greater worry, annoyance, or upset.

**Table 3:** Standard GHAPB goals

Standard GHAPB Goal	Does this situation happen in your life? *N* (% yes)	How much difficulty do you have in this situation? ^a^ Mean (SD) Median (min-max)	How much does any difficulty in this situation worry, annoy or upset you? ^b^ Mean (SD) Median (min-max)
Listening to the TV when the volume is adjusted for others	215 (82.1%)	3.0 (0.7) 3.0 (1.0–5.0)	2.7 (0.9) 3.0 (1.0–5.0)
Having a conversation with one person in quiet	231 (88.2%)	2.0 (0.7) 2.0 (1.0–4.0)	2.1 (1.0) 2.0 (1.0–5.0)
Having a conversation on a busy street or in a shop	229 (87.4%)	3.0 (0.7) 3.0 (1.0–5.0)	2.7 (0.9) 3.0 (1.0–5.0)
Having a conversation with several people in a group	251 (95.8%)	2.9 (0.7) 3.0 (1.0–5.0)	2.8 (1.0) 3.0 (1.0–5.0)

^a^ 1 = no difficulty; 5 = cannot manage

^b^ 1 = not at all; 5 = very much indeed

Participants’ aggregated responses to their two user-nominated goals are shown in Table 4. Participants noted a mean of 3.2 for both user-nominated goals 1 and 2 on a 1 to 5 scale when asked how much difficulty they have in their nominated situation. Participants indicated a mean of 3.2 and 3.3 for user-nominated goals 1 and 2, respectively, on a 1 to 5 scale when asked how much any difficulty in the situation worried, annoyed, or upset them.

**Table 4:** User-Nominated goal responses

User-Nominated Goal	How much difficulty do you have in this situation? ^a^ Mean (SD) Median (min-max)	How much does any difficulty in this situation worry, annoy or upset you? ^b^ Mean (SD) Median (min-max)
User-nominated goal 1	3.2 (0.7)	3.2 (0.9)
	3.0 (1.0–5.0)	3.0 (1.0–5.0)
User-nominated goal 2	3.2 (0.7)	3.3 (0.9)
	3.0 (2.0–5.0)	3.0 (1.0–5.0)

^a^ 1 = no difficulty; 5 = cannot manage

^b^ 1 = not at all; 5 = very much indeed

The complete results from categorization of user-nominated goals into themes are shown in Table 5. The most common themes for participants’ user-nominated goals were having a conversation with several people in a group (standard GHABP goal 4, 80/501, 16.0%), challenges with hearing in background noise (73/501, 14.6%), and listening when unable to see the speaker’s mouth (57/501, 11.4%). Themes covered by standard GHABP goals 1, 2, 3, and 4 were seen in 10.0%, 2.4%, 8.0%, and 16.0%, respectively, of user-nominated goals.

**Table 5:** Characteristics of User-Nominated goals

Description	*N* (%)
Total user-nominated goals	501 (100%)
Themes*:	
Listening to the TV when the volume is adjusted for others (standard Glasgow goal 1)	50 (10.0%)
Having a conversation with one person in quiet (standard Glasgow goal 2)	12 (2.4%)
Having a conversation on a busy street or in a shop (standard Glasgow goal 3)	40 (8.0%)
Having a conversation with several people in a group (standard Glasgow goal 4)	80 (16.0%)
Listening when unable to see the speaker’s mouth	57 (11.4%)
Using technology to listen to other people or content (video conferencing, phone, speaker phone, headphones, etc.)	44 (8.8%)
Background noise causes challenges hearing	73 (14.6%)
Listening to performances, lectures, or someone talking in a large group	37 (7.4%)
Pitch	15 (3.0%)
Music	14 (2.8%)
Quiet or soft speech	31 (6.2%)
Goal fell under a single theme	302 (60.3%)
Goal fell under multiple themes	75 (15.0%)
Goal not specific enough to be actionable (fell under no themes)	115 (23.0%)
Other: Unable to be categorized (fell under no themes)	9 (1.8%)

*Themes are not mutually exclusive; each user-nominated goal may fit under multiple themes; percentages may not add up to 100%

## Discussion

Our results indicate that the standard GHABP goals apply to a majority of participants, with more than 80% of participants indicating that each standard goal applies to them. For user-nominated goals, a diverse set of themes emerged, with each theme representing less than a quarter of the sample. Notably, the participants nominated hearing goals that they had more difficulty with and were more bothered by, on average, than any of the standard GHABP goals. This further highlights the value of offering user-nominated goals. Approximately a quarter of the user-nominated goals were not specific enough to be actionable in clinical settings (or coded under one or more theme). Some examples of goals lacking specificity include “talking with my family,” “hearing conversation,” and “having to ask people to repeat what they are saying,” as these goals encompass a broad range of hearing environments and scenarios with non-specific reasons for the hearing challenges. This highlights an opportunity to urge patients to be as specific as possible in the GHABP instructions. Additional GHABP instructions urging patients to describe their hearing challenges in more detail may be useful. For example, the revised instructions may encourage patients to nominate “new specific situations, environments, or types of sounds” which are important for their hearing goals. Patients could potentially also be provided a list of common themes in hearing goals to serve as a starting point. Many participants indicated the importance of hearing in situations with background noise, either alone or as part of a goal falling under multiple themes. Additionally, many participants indicated a goal of hearing in restaurants. While this is arguably encompassed by standard GHABP goal 3 (having a conversation on a busy street or in a shop), its frequent inclusion as a user-nominated goal suggests participants interpreted restaurants to be distinct from “a shop.” Modification or clarification of the existing GHABP to encompass restaurants could provide clarity on the degree of benefit patients are receiving from hearing aids. One option would be to modify the standard GHABP goal 3 to a more generalized scenario that explicitly mentions background noise and common settings. For example, “having a conversation in a setting with background noise, such as on a busy street, in a shop, at a restaurant, or at a sporting event.”

Another frequently reported theme (57/501, 11.4% of goals) was listening when unable to see the speaker’s mouth. This included conversations with others who are facing away from the listener, in another room from the listener, or wearing a face covering. The importance of this goal is consistent with a prior study that showed patients with hearing loss relied heavily on lip-reading for speech perception, especially when not using a hearing aid [17]. It is important to note, however, that the difficulty with hearing in these situations may also be the result of deterioration of speech quality due to physical barriers to sound and room acoustics, and the description of being unable to see the speaker’s mouth may be an oversimplification.

Many participants also reported the goal of listening when using technology (44/501, 8.8% of goals) such as video conferencing, talking on the phone, or using headphones. This coincides with a 2020 study that showed the importance of communication with technology in quality of life [11]. We suspect these goals were less important to adults with hearing loss at the time of GHABP development, but they will continue to grow in importance due to the lasting impact of the COVID-19 pandemic and ongoing changes in technology and societal norms.

We note that the study enrollment period coincided with part of the COVID-19 pandemic. As a result, there may have been an increased number of user-nominated goals that were driven by hearing needs during a time of stay-at-home orders and universal masking requirements. Nevertheless, while some of these restrictions during the pandemic may have been eased in subsequent years, there is likely still a permanent impact of the COVID-19 pandemic on hearing goals. One such impact is the more widespread use of video conferencing; a study examining two popular video conferencing applications, Zoom and Microsoft Teams, found that the markets for these applications stabilized in late 2020 at higher level than pre-pandemic, though unsurprisingly lower than their peak in March 2020 [18]. Another impact is the use of face coverings, which have been shown to decrease speech intelligibility [19]. While the use of face coverings may not be required in all settings anymore, they may be required in certain environments such as healthcare facilities and have become more normalized during periods of illness or simply as a protective measure. Therefore, it is likely individuals would continue to encounter similar challenges with hearing even after the height of the COVID-19 pandemic. It is difficult to know how trends in video conferencing and face coverings will continue to evolve in the future, and thus changes to the GHABP based on these results alone could introduce bias.

The responses to the four standard GHABP goals, showing that more than 80% of participants encounter each of the scenarios in their life, indicate that these goals remain valuable for understanding patients’ hearing quality of life. Thus, we do not suggest removing any existing standard GHABP goals. We do, however, note that many user-nominated goals in our study fall under the same themes as the four standard GHABP goals. For example, 10% of user-nominated goals related to listening to the TV when the volume is adjusted for others, which is the first standard goal measured in the GHABP. Our results indicate that user-nominated goals can often be redundant with standardized goals. The user-nominated goals portion of the GHABP often takes the longest to complete. Replacing some of the user-nominated goals with standard goals that encompass the range of hearing goals deemed important by many adults with hearing loss could be a useful avenue for capturing additional standardized information in a clinical trial setting. This is especially valuable as demand for healthcare resources outpaces supply, and healthcare workers’ time becomes an increasingly valuable commodity.

As goal attainment scaling gains momentum in clinical trials, there may be opportunities to incorporate one of the key strengths of goal attainment scaling – the training offered to clinicians – to assist patients in nominating specific, actionable goals that do not overlap [20, 21].

There are some limitations to this study. First, as mentioned previously, the initial part of the enrollment period for the parent study coincided with the COVID-19 pandemic, reducing generalizability to non-pandemic times. Second, interpretation of user-nominated goals could be subject to some degree of variability. This is because participants did not always indicate what specific issues they had with hearing in certain situations; for example, a situation such as “listening in the car” could refer to background noise from the car itself, the inability to see other speakers’ mouths due to the seating arrangement, or a combination. Additional instructions in the GHABP may be helpful in eliciting more specific user-defined goals. Alternatively, it may help to have clinicians probe patients for more details regarding nominated goals. Third, our cohort consisted largely of English-speaking participants, reducing generalizability. Finally, our study cohort was derived from individuals who agreed to participate in a clinical trial examining the use of one versus two hearing aids; while all these individuals sought and initiated care for their hearing on their own, it is possible our sample may differ in some ways from the general population of patients seeking hearing healthcare or enrolled in other trials. Nevertheless, the multi-institutional nature of this study does improve the generalizability of our results.

## Conclusions

The existing standard goals measured by the GHABP show continued utility in assessing hearing quality of life in adults with hearing loss. Using themes found when analyzing user-nominated goals from the GHABP, we suggest that the addition of standard goals to encompass as many of those themes as possible could improve the utility of the measure for fully understanding the impact of hearing loss for individual patients.

## Data Availability

The datasets used and/or analyzed during the current study are available from the corresponding author on reasonable request.

## Appendix. Inclusion/Exclusion criteria for overarching study

### Inclusion criteria

1. 50 years of age or older
2. Ability to read and understand English
3. Mild to moderate sensorineural hearing loss (defined by a pure-tone average at 500, 1000, and 2000 Hz of *≤* 55 dB HL in each ear, and the 3000 Hz and 4000 Hz threshold *≤* 80 in each ear), based on a hearing test obtained within the last 6 months by a licensed audiologist.
4. Symmetrical hearing loss defined by < 20 dB difference between the pure-tone average of 500, 1000, and 2000 Hz between ears)
5. Interested in purchasing hearing aids, but is open minded about trying one or two hearing aids
6. No prior hearing aid use longer than 3 months (as documented via self-report)
7. Adequate literacy to complete questionnaires
8. Willing to purchase study-specific hearing aid(s)
9. Access to a smart phone (to receive text messages to link to EMA survey)
10. Has access to internet and ability to receive emails for survey completion

### Exclusion criteria

1. Current concerns for middle ear pathology (e.g., air bone gap of *≥* 15 dB at 2 consecutive octave frequencies in either ear)
2. Current, unresolved concerns for retrocochlear pathology in the opinion of the PI, audiologist, or ENT provider
3. Severe tinnitus as the reason for seeking amplification
4. Co-morbid condition that would interfere with participation in the study in the opinion of the PI, audiologist, or ENT provider
5. History of fluctuating hearing loss

## References

[1] Lin FR, Niparko JK, Ferrucci L (2011) Hearing loss prevalence in the united States. Arch Intern Med 171(20):1851–1853. 10.1001/archinternmed.2011.506 22083573 10.1001/archinternmed.2011.506 PMC3564588

[2] Li C-M, Zhang X, Hoffman HJ, Cotch MF, Themann CL, Wilson MR (2014) Hearing impairment associated with depression in US adults, National health and nutrition examination survey 2005–2010. JAMA Otolaryngol Head Neck Surg 140(4):293–302. 10.1001/jamaoto.2014.42 24604103 10.1001/jamaoto.2014.42 PMC4102382

[3] Mick P, Kawachi I, Lin FR (2014) The association between hearing loss and social isolation in older adults. Otolaryngol Head Neck Surg 150(3):378–384. 10.1177/0194599813518021 24384545 10.1177/0194599813518021

[4] Lin FR, Ferrucci L, Metter EJ, An Y, Zonderman AB, Resnick SM (2011) Hearing loss and cognition in the Baltimore longitudinal study of aging. Neuropsychology 25(6):763–770. 10.1037/a0024238 21728425 10.1037/a0024238 PMC3193888

[5] Contrera KJ, Betz J, Li L, Blake CR, Sung YK, Choi JS, Lin FR (2016) Quality of life after intervention with a cochlear implant or hearing aid. Laryngoscope 126(9):2110–2115. 10.1002/lary.25848 26775283 10.1002/lary.25848 PMC4947575

[6] Livingston G, Sommerlad A, Orgeta V, Costafreda SG, Huntley J, Ames D, Ballard C, Banerjee S, Burns A, Cohen-Mansfield J, Cooper C, Fox N, Gitlin LN, Howard R, Kales HC, Larson EB, Ritchie K, Rockwood K, Sampson EL, Samus Q, Schneider LS, Selbæk G, Teri L, Mukadam N (2017) Dementia prevention, intervention, and care. Lancet 390(10113):2673–2734. 10.1016/s0140-6736(17)31363-6 28735855 10.1016/S0140-6736(17)31363-6

[7] Yeo BSY, Song HJJMD, Toh EMS, Ng LS, Ho CSH, Ho R, Merchant RA, Tan BKJ, Loh WS (2023) Association of hearing aids and cochlear implants with cognitive decline and dementia: A systematic review and Meta-analysis. JAMA Neurol 80(2):134–141. 10.1001/jamaneurol.2022.4427 36469314 10.1001/jamaneurol.2022.4427 PMC9856596

[8] Gatehouse S (1999) Glasgow hearing aid benefit profile: derivation and validation of a Client-centered outcome measure for hearing aid services. J Am Acad Audiol 10(02):80–103. 10.1055/s-0042-1748460

[9] Edgman-Levitan S, Schoenbaum SC (2021) Patient-centered care: achieving higher quality by designing care through the patient’s eyes. Isr J Health Policy Res 10(1):21. 10.1186/s13584-021-00459-9 33673875 10.1186/s13584-021-00459-9 PMC7934513

[10] Kuipers SJ, Cramm JM, Nieboer AP (2019) The importance of patient-centered care and co-creation of care for satisfaction with care and physical and social well-being of patients with multi-morbidity in the primary care setting. BMC Health Serv Res 19(1):13. 10.1186/s12913-018-3818-y 30621688 10.1186/s12913-018-3818-y PMC6323728

[11] Dixon PR, Feeny D, Tomlinson G, Cushing S, Chen JM, Krahn MD (2020) Health-Related quality of life changes associated with hearing loss. JAMA Otolaryngol Head Neck Surg 146(7):630–638. 10.1001/jamaoto.2020.0674 32407468 10.1001/jamaoto.2020.0674 PMC7226291

[12] Smith SL, Ricketts T, Eberts S, Coles T, Bettger J, Francis H, Kilpatrick K, Peskoe S, Rockhold F, Walker A (2024) Randomized controlled trial methodology to examine unilateral vs bilateral fittings. [Poster Abstract] American Auditory Society, Socttsdale, Arizona

[13] Harris PA, Taylor R, Minor BL, Elliott V, Fernandez M, O’Neal L, McLeod L, Delacqua G, Delacqua F, Kirby J, Duda SN (2019) The REDCap consortium: Building an international community of software platform partners. J Biomed Inf 95:103208. 10.1016/j.jbi.2019.103208 10.1016/j.jbi.2019.103208 PMC7254481 31078660

[14] Harris PA, Taylor R, Thielke R, Payne J, Gonzalez N, Conde JG (2009) Research electronic data capture (REDCap)—A metadata-driven methodology and workflow process for providing translational research informatics support. J Biomed Inf 42(2):377–381. 10.1016/j.jbi.2008.08.010 10.1016/j.jbi.2008.08.010 PMC2700030 18929686

[15] National Library of Medicine. NCT04739436 (2024) Evaluation of unilateral vs bilateral hearing aids for the treatment of age-related hearing loss. Available at: https://clinicaltrials.gov/study/NCT04739436. Accessed September 9

[16] Cox RM, Alexander GC (1995) The abbreviated profile of hearing aid benefit. Ear Hear 16(2):176–186 7789669 10.1097/00003446-199504000-00005

[17] Bannwart Dell’Aringa AH, Satico Adachi E, Dell’Aringa AR (2007) Lip reading role in the hearing aid fitting process. Braz J Otorhinolaryngol 73(1):95–99. 10.1016/S1808-8694(15)31129-0 17505606 10.1016/S1808-8694(15)31129-0 PMC9443519

[18] Tudor C (2022) The impact of the COVID-19 pandemic on the global web and video conferencing SaaS market. Electronics 11(16):2633

[19] Bottalico P, Murgia S, Puglisi GE, Astolfi A, Kirk KI (2020) Effect of masks on speech intelligibility in auralized classrooms. J Acoust Soc Am 148(5):2878. 10.1121/10.0002450 33261397 10.1121/10.0002450 PMC7857496

[20] Logan B, Jegatheesan D, Viecelli A, Pascoe E, Hubbard R (2022) Goal attainment scaling as an outcome measure for randomized controlled trials: a scoping review. BMJ Open 12(7):e063061. 10.1136/bmjopen-2022-063061 35868829 10.1136/bmjopen-2022-063061 PMC9316030

[21] Patient-Focused Drug Development: Incorporating Clinical Outcome Assessments into Endpoints for Regulatory Decision-Making (2023) (Department of Health and Human Services Food and Drug Administration, Silver Spring. https://www.fda.gov/regulatory-information/search-fda-guidance-documents/patient-focused-drug-development-incorporating-clinical-outcome-assessments-endpoints-regulatory. Accessed 23 March 2025

